# RNA sequencing data of Notch ligand treated human dental pulp cells

**DOI:** 10.1016/j.dib.2018.01.058

**Published:** 2018-01-31

**Authors:** Jeeranan Manokawinchoke, Praphawi Nattasit, Tanutchaporn Thongngam, Prasit Pavasant, Kevin A. Tompkins, Hiroshi Egusa, Thanaphum Osathanon

**Affiliations:** a Excellence Center in Regenerative Dentistry, Faculty of Dentistry, Chulalongkorn University, Bangkok 10330, Thailand; b Department of Anatomy, Faculty of Dentistry, Chulalongkorn University, Bangkok 10330, Thailand; c Office of Research Affairs, Faculty of Dentistry, Chulalongkorn University, Bangkok 10330, Thailand; d Division of Molecular and Regenerative Prosthodontics, Tohoku University Graduate School of Dentistry, Sendai 980-8575, Japan; e Craniofacial Genetics and Stem Cells Research Group, Faculty of Dentistry, Chulalongkorn University, Bangkok 10330, Thailand

## Abstract

Indirect immobilized ligand has been shown as an effective technique to activate Notch signalling *in vitro.* The data presented in this article are related to the published article entitled “Indirect immobilized Jagged1 suppresses cell cycle progression and induces odonto/osteogenic differentiation in human dental pulp cells” (Manokawinchoke et al. 2017) [1]. This data article describes gene expression in indirect immobilized Jagged1 treated human dental pulp cells (hDPs) using high throughput RNA sequencing technique. These data are valuable to analyze the regulation of Notch signalling in hDPs for understanding its molecular mechanism(s). Raw RNA sequencing data were deposited in the NCBI Sequence Read Archive (SRP100068) and NCBI Gene Expression Omnibus (GSE94989).

**Specifications Table**

**Table:** 

**Subject area**	Biology
**More specific subject area**	Dental pulp biology
**Type of data**	FASTQ file, Tables, Figures
**How data was acquired**	High throughput RNA sequencing
**Data format**	Raw data
**Experimental factors**	Human dental pulp cells were seeded on an indirect immobilized Jagged1 surface.
**Experimental features**	Human dental pulp cells were seeded on an indirect immobilized Jagged1 surface for 24 h and cells on hFc immobilized surface were employed as the control. Total RNA was isolated and mRNA libraries were prepared. RNA sequencing was performed using NextSeq. 500 (Illumina).
**Data source location**	Bangkok, Thailand
**Data accessibility**	Raw data were deposited at NCBI Sequence Read Archive (SRP100068) and NCBI Gene Expression Omnibus (GSE94989).
	https://www.ncbi.nlm.nih.gov/sra?term=SRP100068
	https://www.ncbi.nlm.nih.gov/geo/query/acc.cgi?acc=GSE94989

**Value of the data**

• Differentially expressed genes could be extensively investigated to elucidate the role of Jagged1 activated Notch signalling in human dental pulp biology.
• Specific pathway enrichment could be further analysed to clarify signalling interactions in human dental pulp cells.
• Bioinformatic analysis comparing the response of other cells to Jagged1 could be beneficial to elucidate the biological function of Jagged1.

## 1 Data

Notch signalling regulates various cell functions, depending on cell type and stage of differentiation [1], [2], [3], [4]. Notch ligand, Jagged1, expression was noted in the stromal area in the dental pulp after direct pulp capping with calcium hydroxide [5]. The present data presented the gene expression profile of Jagged1 treated hDPs using RNA sequencing analysis (Table 1).

**Table 1:** Information of samples for differential gene expression of RNA sequencing analysis of indirect immobilized Jagged1 treated human dental pulp cells.

**Subject**	**Source**	**Protocol 1**	**Protocol 2**	**Protocol 3**	**Sequencer**	**Read Length (bp)**	**GEO accession number**
Donor 1	Human dental pulp cells	Human Fc immobilization (hFc)	Total RNA extraction	RNA-Seq	Illumina NextSeq. 500	75 reads paired-end	GSM2493825
Donor 1	Human dental pulp cells	Recombinant human Jagged1/Fc immobilization (Jagged1)	Total RNA extraction	RNA-Seq	Illumina NextSeq. 500	75 reads paired-end	GSM2493828
Donor 1	Human dental pulp cells	Pretreatment with a gamma secretase inhibitor before exposing to recombinant human Jagged1/Fc immobilization (Jagged1+DAPT)	Total RNA extraction	RNA-Seq	Illumina NextSeq. 500	75 reads paired-end	GSM2493831
Donor 2	Human dental pulp cells	Human Fc immobilization (hFc)	Total RNA extraction	RNA-Seq	Illumina NextSeq. 500	75 reads paired-end	GSM2493826
Donor 2	Human dental pulp cells	Recombinant human Jagged1/Fc immobilization (Jagged1)	Total RNA extraction	RNA-Seq	Illumina NextSeq. 500	75 reads paired-end	GSM2493829
Donor 2	Human dental pulp cells	Pretreatment with a gamma secretase inhibitor before exposing to recombinant human Jagged1/Fc immobilization (Jagged1+DAPT)	Total RNA extraction	RNA-Seq	Illumina NextSeq. 500	75 reads paired-end	GSM2493832
Donor 3	Human dental pulp cells	Human Fc immobilization (hFc)	Total RNA extraction	RNA-Seq	Illumina NextSeq. 500	75 reads paired-end	GSM2493827
Donor 3	Human dental pulp cells	Recombinant human Jagged1/Fc immobilization (Jagged1)	Total RNA extraction	RNA-Seq	Illumina NextSeq. 500	75 reads paired-end	GSM2493830
Donor 3	Human dental pulp cells	Pretreatment with a gamma secretase inhibitor before exposing to recombinant human Jagged1/Fc immobilization (Jagged1+DAPT)	Total RNA extraction	RNA-Seq	Illumina NextSeq. 500	75 reads paired-end	GSM2493833

## 2 Experimental design, materials and methods

These methods are expanded versions of descriptions in our related work [1].

### 2.1 Cell isolation and culture

Human dental pulp cell isolation protocol was approved by the Human Ethics Committee, Faculty of Dentistry, Chulalongkorn University (Study code HREC-DCU 2016-074). Inform consent was obtained. Teeth scheduling for extraction according to treatment plan (impacted third molars) were collected for cell isolation. Briefly, dental pulp tissues were gently removed and minced. Cell isolation was performed by explant protocol. Cells were maintained in Dulbecco's Modified Eagle's Medium (Gibco, Carlsbad, CA, USA) supplemented with 10% fetal bovine serum (Gibco), 2 mM L-glutamine (Invitrogen, Carlsbad, CA, USA), 100 Units/ml penicillin (Invitrogen), 100 μg/ml streptomycin (Invitrogen), and 250 ng/ml amphotericin B (Invitrogen) at 37 °C in a humidified 5% CO_2_ atmosphere. Cells from passage 3–5 were used in the work.

For Jagged1 treatment, recombinant human Jagged1/Fc fusion protein (10 nM; R&D systems, Minneapolis, MN, USA) was indirectly immobilized on tissue culture surfaces according to a previously published protocol [6]. Cells (at density of 300,000 cells per wells in 6 well-plate) were seeded on Jagged1 immobilized surface for 24 h. The human immunoglobulin G Fc fragment protein (hFc) alone was used as the control. To inhibit Notch signalling, cells were pretreated with (N-[N-(3,5-Diflurophenaacetyl-L-alanyl)]-S-phenylglycine t-Butyl Ester) (DAPT; Sigma, 20 μM) 30 min prior to Jagged1 exposure and further maintained in culture medium for 24 h.

### 2.2 RNA preparation and sequencing

**Fig. 1:**
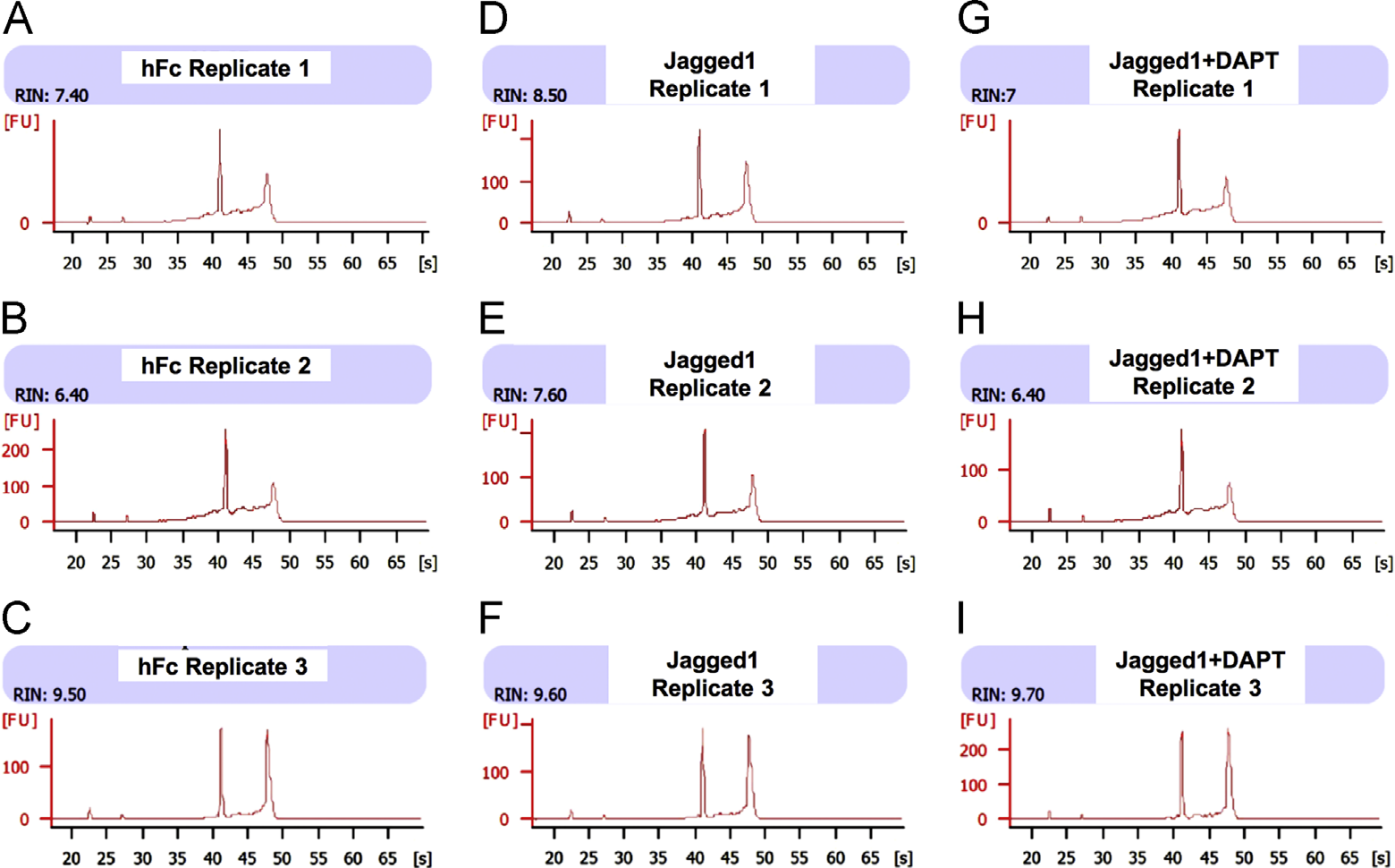
Quality check of input total RNA using the Bioanalyzer. (A-C) hFc replicates; (D-F) Jagged1 replicates; (G-I) Jagged1+DAPT replicates.

RNA preparation, RNA sequencing, and bioinformatics analysis was performed at the Omics Science and Bioinformatics Center, Faculty of Science, Chulalongkorn University. Three biological replicates were employed in each group for RNA sequencing analysis. RNA isolation was performed using an RNeasy kit (Qiagen, Valencia, CA, USA) according to the manufacturer's protocol with DNaseI treatment. RNA was eluted from the column using nuclease free water. The preliminary RNA quality and quantity were evaluated using a Nanodrop instrument. Further, RNA quality was examined using a bioanalyzer (Aligent 2100; Agilent Technologies, Santa Clara, CA, USA). The isolated RNA exhibited an OD260/280 ratio of 2.07–2.11 and the OD260/230 ratio was from 1.75–2.09. The concentration of the isolated RNAs ranged from 214.3–424.7 ng/μl. The RNA quality was further confirmed using a bioanalyzer (Aligent 2100; Agilent Technologies, Santa Clara, CA, USA). The RNA integrity number (RIN) was calculated for each sample. The RIN of all 9 samples was demonstrated and exhibited an acceptable quality of input for sequencing library construction (Fig. 1).

**Fig. 2:**
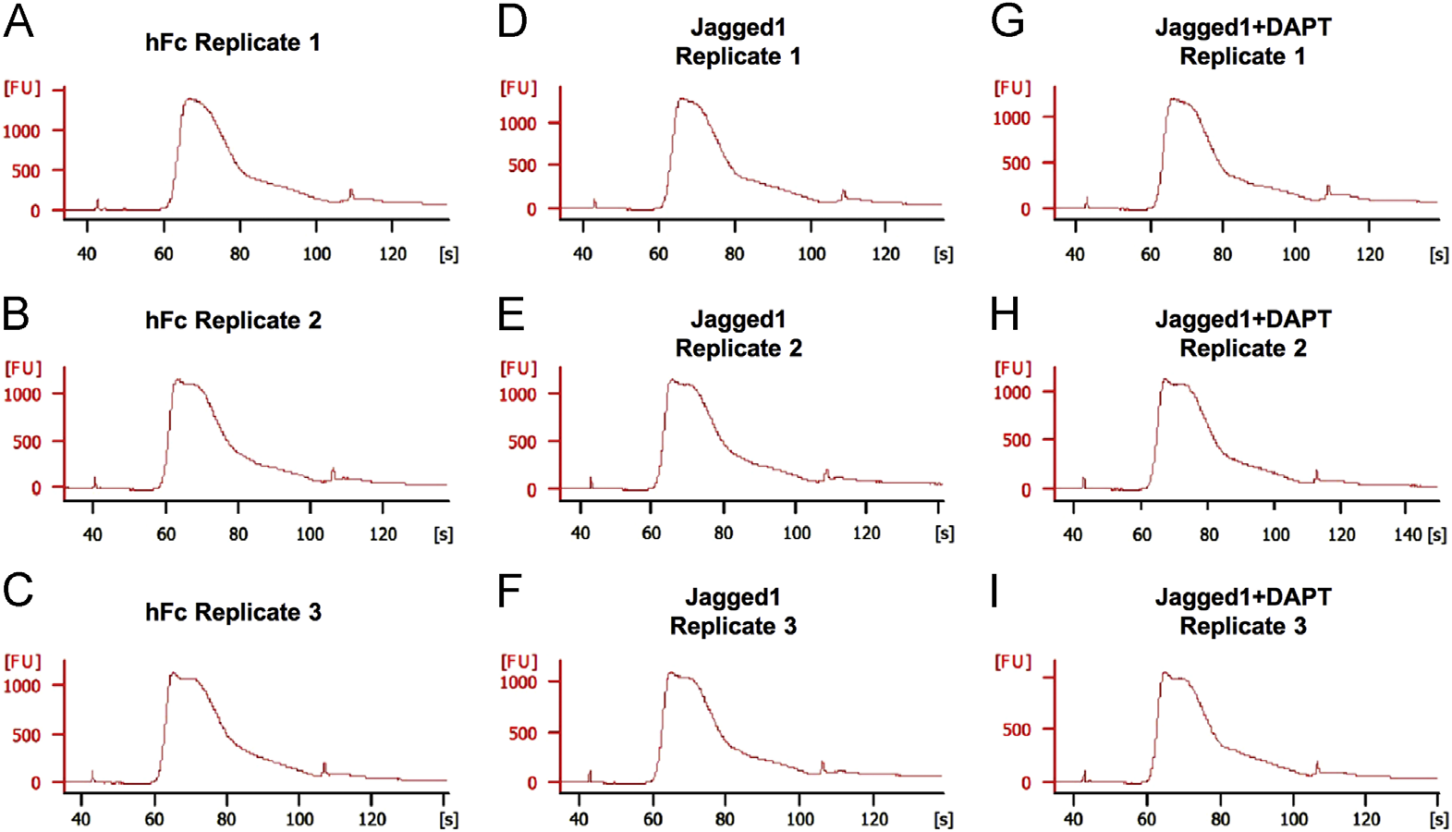
Library quality and size check using the Bioanalyzer. (A-C) hFc replicates; (D-F) Jagged1 replicates; (G-I) Jagged1+DAPT replicates.

Total RNA (1 μg) was used for mRNA library preparation. The TrueSeq mRNA stranded library preparation kit (Illumina, San Diego, CA, USA) was employed. Sequencing library quality was examined using an Agilent 2100 Bioanalyzer (Agilent Technologies) (Fig. 2). The average library size and concentration were determined using a Qubit 3.0 fluorometer (Thermo Fisher Scientific, Waltham, MA, USA) (Table 2).

**Table 2:** Average library size and concentration.

**Sample ID**	**Qubit concentration (ng/µl)**	**Average library size (bp)**
hFc Replicate 1	32.3	267
Jagged1+DAPT Replicate 1	35.9	275
Jagged1 Replicate 1	33.1	252
hFc Replicate 2	34.9	270
Jagged1+DAPT Replicate 2	33.7	300
Jagged1 Replicate 2	34.3	277
hFc Replicate 3	33.1	275
Jagged1+DAPT Replicate 3	33.1	266
Jagged1 Replicate 3	36.2	271

The libraries were pooled at a concentration of 10 nM and the sequencing analysis was performed using the NextSeq. 500 (Illumina).

### 2.3 Quality validation and read mapping

Base calling (https://support.illumina.com/sequencing/sequencing_instruments/nextseq-500.html) and Q scoring was performed by RTA2 software. File conversion and demultiplexing were performed using bcl2fastq software. Read quality was checked, trimmed, and filtered by the FastQC (http://www.bioinformatics.babraham.ac.uk/projects/fastqc/) and FastX Toolkit (at http://hannonlab.cshl.edu/fastx_toolkit/commandline.html). Read mapping was performed against *Homo sapiens UCSC hg38* using the TopHat2 program (https://ccb.jhu.edu/software/tophat/index.shtml). Fragments Per Kilobase of transcript per Million mapped reads (FPKM) estimation of reference genes and transcripts as well as assembly of novel transcripts were performed using Cufflink2 (http://cole-trapnell-lab.github.io/cufflinks/). Variant calling was performed using the Isaac Variant caller.

**Fig. 3:**
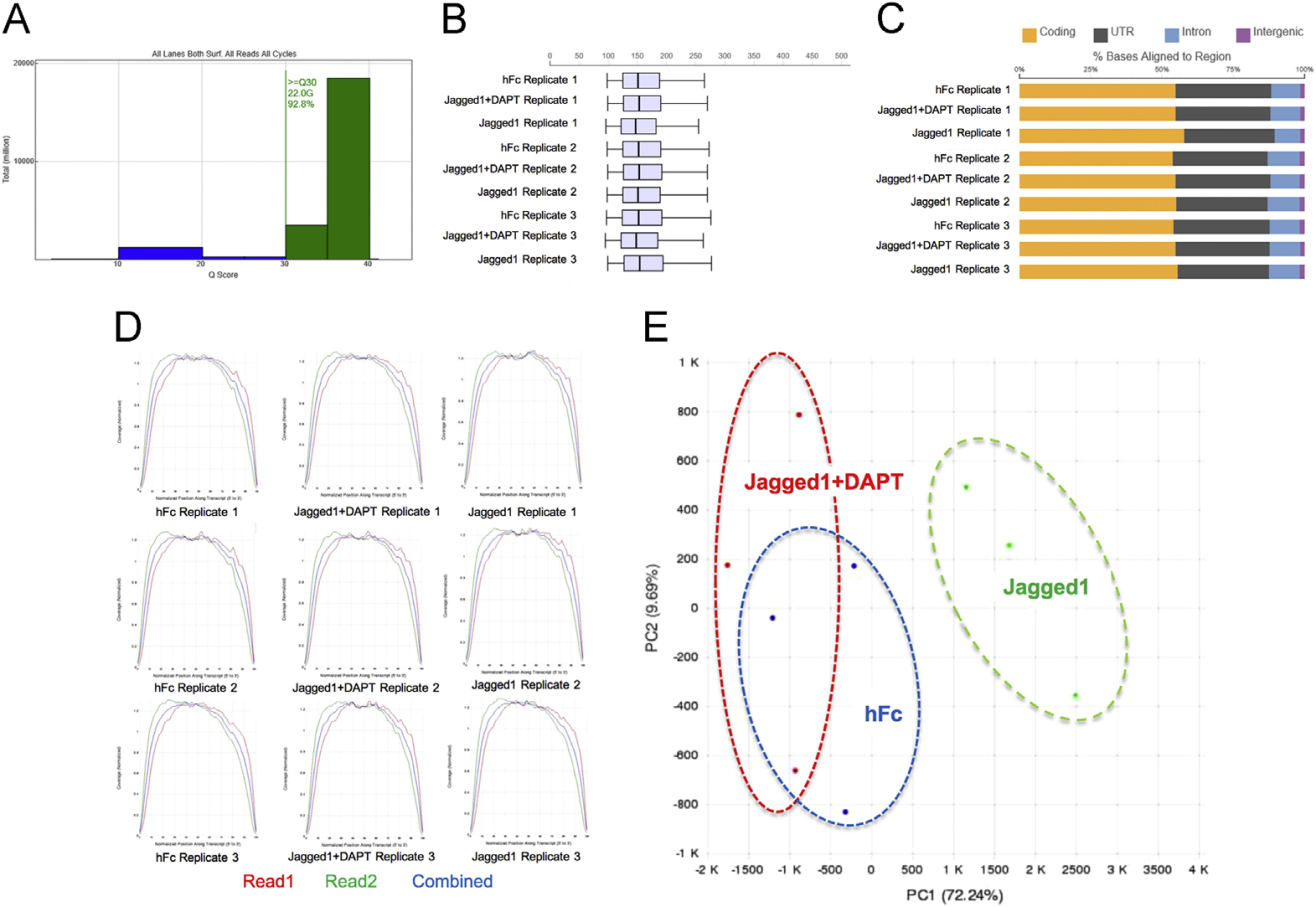
The Q Score Distribution Plot demonstrates the number of bases by quality score of the NextSeq run (A). Insert length distribution (B) and alignment distribution after RNA sequencing alignment are illustrated. Transcript coverage graphs show the coverage of a transcript aligned to a position on sequencing reads (D). Principle component analysis diagram (PCA) was examined to evaluate variance among groups and samples (E).

The NextSeq run yielded 300 million reads (Table 3). Each sample contained roughly 30 million (75 base pair; paired-end) reads. The NextSeq run generated high quality output reads (22.0 Gbp or 92.8% Q30) (Fig. 3A). The base calling error rate was 0.40%. After trimming, approximately 5% of the total reads across all samples were lost. The RNA-Seq alignment summary is shown in Table 4. The insert length distribution and alignment distribution are provided (Fig. 3B). The coverage of a transcript aligned to a position on sequencing reads of all samples is illustrated (Fig. 3C). Transcript coverage graphs show the coverage of a transcript aligned to a position on sequencing reads (Fig. 3D). The principle component analysis diagram was examined to evaluate variance among groups and samples (Fig. 3E).

**Table 3:** NextSeq run summary.

**Read**	**Cluster passing filter (%)**	**Read passing filter (millions)**	**Error rate (%)**	**Q score > 30 (%)**
Read 1 (Forward-end)	91.3	150	0.34	94.1
Read 2 (Reverse-end)	91.3	150	0.45	91.4
Total	91.3	300	0.4	92.8

**Table 4:** RNA-Seq alignment summary.

**Sample ID**	**Number of reads**	**Total aligned (%)**	**Abundant (%)**	**Unaligned (%)**	**Stranded (%)**
hFc Replicate 1	16,535,551	95.15	3.14	4.85	99.75
Jagged1+DAPT Replicate 1	17,210,538	95.45	3.51	4.55	99.73
Jagged1 Replicate 1	14,859,041	93.89	2.25	6.11	99.71
hFc Replicate 2	16,038,419	95.86	3.31	4.14	99.71
Jagged1+DAPT Replicate 2	16,592,411	94.1	3.49	5.9	99.73
Jagged1 Replicate 2	17,819,532	95.5	3.18	4.5	99.71
hFc Replicate 3	15,484,155	93.67	3.22	6.33	99.72
Jagged1+DAPT Replicate 3	16,341,280	94.89	3.37	5.11	99.74
Jagged1 Replicate 3	15,159,403	91.55	2.91	8.45	99.72
